# Design-redesign, implementation, and evaluation of effectiveness of maternal nutrition and responsive parenting program on child development at 2 years of age from rural India: a cluster RCT

**DOI:** 10.3389/fpubh.2023.1165728

**Published:** 2023-11-14

**Authors:** Abhay Gaidhane, Mahalaqua Nazli Khatib, Shital Telrandhe, Manoj Patil, Priti Kogade, Shilpa Gaidhane, Sonali G. Choudhari, Penny A. Holding, Deepak Saxena, Zahiruddin Quazi Syed

**Affiliations:** ^1^Centre of One Health, School of Epidemiology and Public Health, Jawaharlal Nehru Medical College, Datta Meghe Institute of Higher Education and Research, Wardha, Maharashtra, India; ^2^Global Evidence Synthesis Initiative, Division of Evidence Synthesis, Jawaharlal Nehru Medical College, Datta Meghe Institute of Higher Education and Research, Wardha, Maharashtra, India; ^3^Global Health Academy, Centre of Early Childhood Development - Stepping Stones Project, Wardha, India; ^4^Datta Meghe Institute of Higher Education and Research, Wardha, Maharashtra, India; ^5^School of Epidemiology and Public Health, Jawaharlal Nehru Medical College, Datta Meghe Institute of Higher Education and Research, Wardha, Maharashtra, India; ^6^Jawaharlal Nehru Medical College, Datta Meghe Institute of Higher Education and Research, Wardha, Maharashtra, India; ^7^i Health Consortium, Department of Epidemiology, Indian Institute of Public Health, Gandhinagar, Gujarat, India; ^8^South Asia Infant Feeding Research Network (SAIFRN), School of Epidemiology and Public Health, Wardha, Maharashtra, India

**Keywords:** early child development, integrated intervention, nutrition program, responsive parenting, rural India

## Abstract

**Background:**

To promote early childhood development (ECD), we require information not only on what needs to be addressed and on what effects can be achieved but also on effective delivery methods that can be adapted to local context. We describe design, implementation, and evaluation of a complex intervention to strengthen nurturing environment for young children.

**Methods:**

Study participants were pregnant women and their children from birth to 2 years. We used design and redesign, implementation, and evaluation approaches for the study. We co-created curriculum and delivery plan with stakeholders, based on the theoretical framework, findings from formative research, and our preliminary work. We recruited 656 pregnant women and newborns, 326 (49.69%) from intervention and 330 (50.30%) from the control group. We conducted a cluster randomized controlled trial to evaluate the program’s effectiveness. The outcomes of children were assessed at 12 and 24 months.

**Findings:**

At recruitment, study participants from both the study arms were similar in sociodemographic characteristics. We conducted 6,665 home visits, 25 toy-making workshops, and 65 caregiver-meetings. The initial examination of program data revealed gaps in quality and coverage of interventions. The intervention was redesigned based on feedback from stakeholders in community meetings. At recruitment, participants in both study groups had similar socio-demographics. We conducted 6,665 home visits, 25 toy workshops, and 65 caregiver meetings. Initial program data showed intervention quality and coverage gaps, leading to a redesign program based on community and stakeholder feedback. Post-re-designing, session quality improved, with program coverage rising from 32 to 98%. Male participation in home visits increased from 4.3 to 32.65%, and data errors reduced from 270 to 140 per month on average. At 24 months, program showed moderate–mild impact on ECD – cognitive (0.31, 95%CI: 0.13–0.48), language (0.2, 95%CI: 0.01–0.39), and socioemotional-development (0.19, 95%CI: 0.01–0.37), moderate effect on home-environment and mother–child interaction. 96% of women initiated breastfeed within one-hour of delivery, and exclusive-breastfeeding rate of 89.80%.

**Interpretations:**

The study provides an evidence-based community centered ECD curriculum and implementation strategies to enhance service providers, and caregivers’ knowledge and skills for promoting ECD in low-resource settings with the potential to scale within existing Government Program.

**Funding:**

The trial was funded by the Saving Brains Round 5 Initiative of Grand Challenges Canada (Grant no. SB-1707-05084), and we are grateful for their ongoing support through online sessions and orientation workshops. The trial was also supported by the Indian Council of Medical Research (File No: 5/7/1693/CH/Adhoc/RBMCH-2020).

## Introduction

The early years are crucial to ensure that each child reaches their productive and creative potential in adulthood (1, 2). To provide adequate nurturing care, families must address multiple needs for psychosocial stimulation, health care, nutrition, and environmental and economic security (3–7). Evidence of the effectiveness of single-target interventions in the early years of life is available and encouraging. However, information that adequately guides implementing complex programs that address holistic child development is limited.

A Holistic Early Childhood Development (ECD) fosters the overall growth of a child, with the various domains of child development collectively shaping a child’s development and growth. This includes their physical development (i.e., gross and fine motor skills), brain or cognitive development, language development, socio-emotional development and behavioral development.

India’s national Integrated Child Development Service (ICDS) program was initiated in 1975 to tackle child malnutrition and illnesses. ICDS is one of the government’s most extensive and prominent initiatives that offer nutritional supplementation, immunization, height and weight monitoring, and non-formal education to children under six through Anganwadi Centers (AWCs). An Anganwadi Worker (AWW), operating at the grassroots level, is responsible for catering to a thousand population through an Anganwadi Center (AWC), with assistance from the Anganwadi Helper (AWH). However, the recent assessment of the ICDS programs recommended reinforcing the infrastructure, training, and support systems for AWC and staff. The report suggested an adapted curriculum and a framework to oversee the program implementation. Despite the primary emphasis of ICDS being on the early years of life, its efforts primarily revolve around nutritional supplementation and children’s healthcare needs. Unfortunately, the responsive parenting program, vital for fostering early childhood development, is considerably underrepresented within ICDS (8).

In India, 55% of children under 6 months are exclusively breastfed. Although breastfeeding is nearly universal in Maharashtra, only 57% of children under 6 months are exclusively breastfed, as the World Health Organization (WHO) recommends. Encouragingly, 87% of infants are introduced to breastfeeding within the first day of their lives, but it drops to 57% for those who begin breastfeeding within the recommended first hour of life. In the context of child health, the infant mortality rate in Maharashtra in NFHS-4 is estimated at 24 deaths before the age of 1 year per 1,000 live births (9).

Integrating parenting with nutrition interventions blended with traditional community-focused child-rearing approaches for Early Child Development (ECD) are evidence-based practices proven effective for ECD (6, 10–12). To take these complex interventions to scale requires a commitment of resources, often scarce and constantly competing with other demands. To achieve sustainability at scale, detailed evidence is necessary that convinces parents, service providers, policymakers, and the political system of the feasibility and value of the intervention in context, and thus to take on these costs (12–16).

In this paper, we describe the design, implementation, and evaluation of a complex intervention to strengthen the nurturing environment for young children. The evidence for ECD intervention programs is well established; however, a critical design issue for such complex interventions is adequately addressing the sociocultural context and current childcare practices (17). Our proposed family-centered, locally developed intervention aimed to enhance the ICDS services targeted at the 0–2 years of age. The theory of change assumes that the additional components of this study shall enhance responsive parenting competencies and improve children’s developmental trajectory (18, 19).

This study aims to determine the effectiveness of the integrated responsive parenting and nutrition program on child development outcomes in children under 2 years from rural India. The study also reflects upon the cycle of design, implementation, and evaluation using the lens of the Measurement for Change (10, 20) to develop an insight into the path to generating sustainability at scale.

## Methodology

### The context and the target population

The study area was a hard-to-reach rural setting, remotely located in two Blocks of the Wardha and the Nagpur districts in central India. The study villages were in the Forest Buffer Zone, a Tiger Sanctuary. People in the study area had an average annual per-capita income below the state average and worked as unskilled daily wage laborers in forests, farms, or cattle rearing. The traditional socio-cultural customs greatly influence childcare practices in these regions. Availability and accessibility of education, health, and social services for people from these villages are challenging, and access worsens during the rains and summer. Women in these villages are overburdened; as they are traditionally responsible for childcare, they work for income and face gender-specific risks and vulnerabilities. Wages for women are lower than for men. This social and economic distress contributes to challenges to adequate nutrition and caregiving.

## Study design

We evaluated an integrated community-based intervention using a Cluster-Randomized Control Trial (C-RCT) design. We ensured all cluster members received similar interventions. Administratively, districts are subdivided into talukas or blocks in India. One taluka has around five Primary Health Centers (PHCs), and PHCs comprise five to six sub-centers (SCs). Sub-center caters to nearly five to six villages with a population of around 5,000. Each village has an Anganwadi center (AWC) catering to a population of 1,000 in a rural area.

We selected two adjacent blocks in central India, the Seloo and the Hingna blocks. The study team has strong community linkages in these areas, so delivering intervention and data collection in selected blocks was convenient. Since the community volunteers administered the intervention at the village level (Anganwadi center area) and the villages are situated very close to each other, we defined the sub-center as a unit of randomization to minimize the risk of contamination across the intervention and control groups. We used a stratified randomization approach.

## Randomization and masking

**Figure 1:**
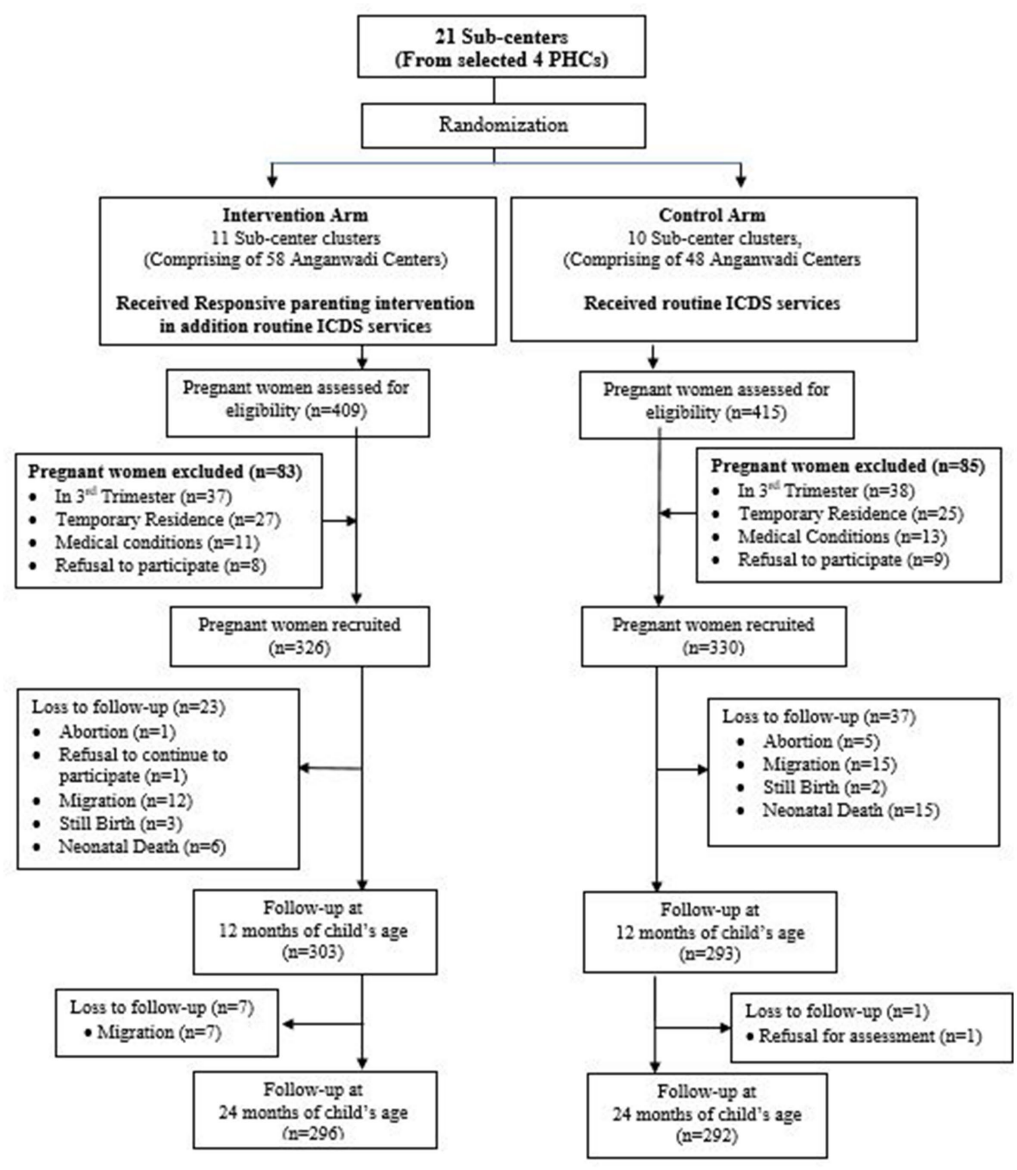
Consort flow chart.

The unit of randomization were sub centers. We randomly selected four PHCs comprising 21 sub-centers (clusters) and 106 villages from the study area. The 21 subcenters were randomly allocated using a random number sequence in the intervention and the control group. The random allocation of clusters were masked for the study team. The intervention group had 11 sub-centers (clusters) comprising 58 villages, and the control had ten sub-centers (clusters), including 48 villages. The intervention clusters received study intervention in addition to the routine ICDS services, while the participants from the control clusters received the routine ICDS services as part of the study (Figure 1).

## Participants

The participants were pregnant women and their children from birth to 2 years residing in selected villages. We recruited pregnant women in the first or second trimester of pregnancy, permanently residing in study areas after informed written consent. High-risk pregnancies have the potential to introduce confounding factors into the study, making it challenging to determine the true impact of the intervention. By excluding high-risk cases, we aimed to enhance the quality and reliability of the data, thereby reducing the likelihood of bias that could affect the child outcome assessment. Additionally, this exclusion not only prioritized the safety of high-risk pregnant women by referring them to specialized healthcare facilities but also enhanced the generalizability of our findings, increasing their relevance to a wider range of expectant mothers. We recruited the participants over a period of 6 months, during which we provided counseling to pregnant women and their families, explaining the nature and purpose of the study.

### From design to implementation to evaluation


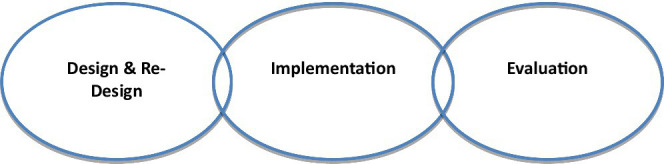


## Intervention design and development

We conducted formative research and collected data on the household, community, and environmental factors related to caregiving practices that influence the growth and development of children under 3 years through three group discussions at village levels and six interviews of Anganwadi workers, caregivers and other stakeholders. The formative research also explored the underlying beliefs and attitudes around childcare practices, nutrition practices, breastfeeding and complementary feeding practices, household decision-making, income levels, cultural traditions, and social norms around childcare. Additionally, the study team conducted three community meetings to understand the sociocultural and childcare practices and specific challenges to access health and childcare services. In these meetings, the study team informed the community about the proposed Project and sought their consent for intervention. We engaged the community at all levels including intervention design, implementation, and program monitoring. During these community meetings, through a collaborative approach, community members collectively selected Balsakhi- a community volunteer from the village to implement the intervention. The team also collected data on nutrition practices, including breastfeeding and complementary feeding practices, household decision-making, income levels, and cultural traditions and social norms around childcare.

The development of a responsive parenting package drew its theoretical framework from Vygotsky’s Zone of Proximal Development (ZPD). The ZPD is a theoretical framework that focuses on the difference between a learner’s actual developmental level and their potential developmental level when provided with appropriate guidance and support. This concept involves nurturing independent actions, skills, and knowledge by offering the necessary scaffolding (11–13). Based on the theoretical framework, the findings from formative research, and our preliminary work (17, 21), we identified six areas of parental competencies, namely (1) shelter and nurturing care, (2) food and nutrition, (3) protection and discipline, (4) socio-emotional learning, (5) health, and (6) early childhood education.

The study team co-created a curriculum comprising locally/culturally appropriate play-based activities covering these six groups’ identified parental competencies. Community members, Anganwadi workers and supervisors, schoolteachers/early childhood educators, and other stakeholders participated in the curriculum development process. The enhanced nutrition program included a nutrition demonstration center and locally and culturally relevant recipes for pregnant women and infant and young child complementary feeding. The program linked the study participants with local Anganwadi centers to provide them with supplementary nutrition support. The program also integrated monitoring, evaluation, and learning (MEL) processes to track progress, direct adaptation for improved efficiency, and evaluate impact. Thus, the intervention package comprised age-appropriate content, curriculum, and necessary tools and materials. We designed tools to enable parents to use them with their children to promote their children’s cognitive, language, socioemotional, and physical development at 2 years of age.

### Implementation

The intervention delivery approach was community-driven, as community-based programs are an essential service delivery approach for early childhood intervention in under-resourced and developing contexts. Community-based implementation provides scope for identifying and analyzing specific community issues and prioritizing, designing, and managing activities at the local level (22). Our delivery approach focuses on interactive discussions adapted to the family’s needs and the appropriate use of tools. Delivery was also intended to enable a Cluster Randomized Controlled Trial (cRCT) that assessed effectiveness and feasibility.

#### The delivery team

A ‘Balsakhi’ community volunteer delivered the intervention. Balsakhi is a local Marathi word, and it means ‘child’s friend.’ Balsakhi were women from the village, preferably married, with some education (able to read and write in the local language), and willing to volunteer for 2–3 h daily. We selected one Balsakhi for each study village by conducting interviews and administering written examinations to evaluate basic skills and trained them.

The initial training spanned 3 days, certifying participants for conducting home visits, group sessions, and community workshops. During the Project’s initial phase, the Project Research Associates accompanied each Balsakhi to provide initial support. The Balsakhi received refresher training on a one-to-one basis whenever required. The Balsakhi were mentored and supported by Anganwadi workers from the same village to deliver the responsive parenting intervention in the respective villages. Competencies of Balsakhis are assessed through regular supervision, observations of their interactions with children and parents, and periodic evaluations to gauge their knowledge and performance in providing childcare and early education services. Each Balsakhi received a monthly honorarium for their services.

#### Home visiting program

The focus was on the early initiation of breastfeeding, exclusive breastfeeding, and the appropriate introduction of complementary feeding. The Balsakhi through 44 home visits delivered the intervention over 24 months. We trained the Balsakhi to deliver the session interactively, using activities and reflective practice. We equipped the Balsakhis with guidebooks, manuals, posters, flyers, flip charts, books, and toys to support session delivery. In addition to the material provided to Balsakhi, she was encouraged to use everyday household items in sessions. The trained and motivated Balsakhi to avoid being directive, adapt to the family’s needs, and keep the family actively engaged in developing their knowledge and skills as responsive parents.

#### Community workshops

We organize a community workshop to support caregivers in preparing ECD play materials using accessible materials in alternate months. The workshops also demonstrated how to use the toys/play material, what development domain they stimulate, and what to observe while using the play material. The emphasis focused on health and safety issues when using the play materials. We provided the caregivers with a booklet and activity cards to guide them in toy making and play activities.

#### Community group meetings

We conducted monthly group meetings to create an enabling environment for ECD at the village level by intentionally building partnerships to support this process. The research assistants and Balsakhi coordinated these community meetings, inviting all stakeholders: Anganwadi workers, caregivers/parents, panchayat leaders, schoolteachers, volunteers, and the research team. We used a collaborative learning and sharing approach by inviting caregivers and parents to share their experiences around ECD and asking the larger group to reflect on those experiences. Based on the data, the stakeholders discussed the implementation progress and formulated a plan to improve intervention delivery and coverage. A total of 58 sessions were conducted in all the intervention villages.

#### Nutrition intervention

We created one demonstration center in each PHC area. The caregivers/mothers were invited to this demonstration center monthly for interaction and discussions on creating recipes for pregnant mothers and young children. In addition to these activities, we created a nutrition garden in households, wherever at least 200 square feet of space was available. Eighteen kitchen gardens were set up in the intervention villages. The Project team provided the seeds and saplings, whereas the household members sowed the seeds and maintained the nutrition garden. More preference was given to foods that were locally consumed.

#### Integrated MEL

Each child received the program for 24 months. To ensure the coverage and fidelity of the intervention, we analyzed intervention data weekly and tracked information on utilization and engagement, as well as the quality of interactions. Data on attendance, meeting schedules, and using the intelligent register application to give personalized feedback to parents were collected on a PC tablet application. Program supervisors used this application to monitor activity, give feedback and initiate discussions with the delivery team to improve the quality and coverage of the intervention (14).

## Complementing the government’s ICDS program

Every participant in the study was enrolled in the standard services offered by Anganwadi centers, where children under six receive non-formal education, nutrition supplementation, and growth monitoring. Furthermore, the intervention group benefited from responsive parenting and enhanced nutrition initiatives delivered through home visits, group sessions, and community workshops. This intervention strategy synergizes with the existing components of the ICDS program, introducing an adaptable community-focused, responsive parenting curriculum for children under 2 years of age. The ICDS program’s Anganwadi Workers played a supportive role, guiding the Balsakhi and aiding in addressing challenges related to intervention delivery.

## Impact assessment

### Outcome measurement

At recruitment, the study team collected household sociodemographic data and antenatal characteristics of pregnant women using the Government of India Demographic Health Survey (DHS) tool (9). Field staff received training and certification in completing DHS forms at recruitment and baseline data collection. We administered a battery of tools to assess child development outcomes at 12 months and 24 months of age. We chose assessment tools previously used in low-middle-income country settings (27). The study team adapted tools to the local context, translated them from English to Marathi (the local language), and back-translated them into English. Language experts validated the translated tools.

Developmental Milestones Checklist (DMC) is a reliable and sensitive tool for evaluating motor, language, and personal-social development (23, 24). We considered the gross score of DMC tool for the cognitive development of children under two years. Profile of Socio-Emotional Development (PSED) assesses children’s social and emotional development through observation and parental reports (25). PSED tool was adapted to the local context and incorporated culturally and socially relevant items. The adapted home environment was assessed at baseline and the endpoint using the Infant-Toddler Home Inventory (26–28). The quality of mother–child interaction was evaluated using the Observation of the Mother–Child Interaction (OMCI) (29), and parent behaviors, parental knowledge, and skills for ECD were assessed using the Photostory approach and a parental quiz (17, 21). We used standard established protocols of WHO for anthropometric assessments (30).

The field staff who administered assessment tools received training and certification (17). The outcome assessors were masked to the intervention. Assessors also worked independently with the community volunteer and Anganwadi workers who delivered the intervention. To reduce familiarity with households and caregivers, the research team randomly rotated the assessor team across clusters. The assessors were instructed to refrain from inquiring about the families’ intervention status. Additionally, inter-rater reliability testing was conducted to ensure data quality and consistency. The reliability coefficient for the Development Milestone Checklist (DMC) scale, Observation of Mother–Child Interaction (OMCI) tool, Profile of Socio-emotional Development (PSED), and Home Scale Coding (HSC) was 0.875, 0.691, 0.673 and 0.759, respectively. The data team developed an XLS file for all devices and then imported those files to Open Data Kit (ODK). Data collection tools were then imported into an Android Tablet-PC. The app had in-built quality checks that monitored score distributions and missing values/data. Using a tablet PC for data collection, we trained evaluators in the ODK process.

At recruitment, we captured the household information and mothers’ maternal characteristics using the Demographic Health Survey tool of the Government of India. At 12 months and 24 months, we assessed all child development outcomes (primary outcomes) – Physical, cognitive, language, and socioemotional development. We also evaluated mother–child interaction and home environment at 12 and 18 months of interventions.

### Sample size

We aimed to detect differences of 0.3SD between the intervention and the control groups. The preliminary data from the study area calculated the child development score of 67 in the cognitive domain. We assume the Intra-cluster Correlation Coefficient (ICC) of child development as 0.02, with the average number of pregnancies per cluster per year as 30, resulting in the design effect 1.58. Hence, to detect the desired improvement of 0.3SD in development score in the intervention group, with 95% confidence and 80% power, a total sample size of 452 mother–child dyads. Based on the previous experience, we accounted for a 20% loss to follow-up. Thus, the final sample size is 542 from 21 clusters, 271 in each group. However, from an ethical perspective, we enrolled all eligible participants fulfilling the inclusion criteria from the intervention and the control clusters in the study.

### Analysis

We used STATA version 14 for analysis. We compared household and sociodemographic data from the intervention and the control group to ensure the robustness of the randomization process and to examine the characteristics of participants lost to follow-up. We used the intention-to-treat analysis to compare the child outcomes between the intervention and control groups at 12 months and 24 months by a mixed effect regression model adjusted for cluster and assessors and controlled for potential confounders (mothers’ education, child sex, wealth index, total family members). The mean child development scores for all domains are presented with a 95% confidence interval, considering *p* < 0.05 for the statistical significance. We also estimated the intervention’s effect size at 12 and 24 months as ‘Cohens *d*’, as the difference in the adjusted mean between the intervention and the control group divided by the pool SD.

#### Registration

We registered the trial with clinical trial registry of India under the CTRI Number: CTRI/2017/05/008553 on 15/05/2017. The Institutional Ethics Committee of Datta Meghe Institute of Medical Sciences (Deemed to be University) approved the trial vide letter with Ref no: DMIMS (DU)/IEC/2017–18/6306 dated 27.03.2017.

## Results

### Recruitment and engagement

We assessed 824 participants for eligibility and recruited 656 (79.61%) eligible women in their second trimester of pregnancy and their newborns. The intervention group had 326 (49.69%), and the control group had 330 (50.30%) participants at the enrolment. At 24 months, the study endpoint, 68 (10.36%) participants lost to follow-up.

Table 1 provides characteristics of study participants from the intervention and the control arm at enrolment. Study participants’ sociodemographic characteristics were comparable between the two groups, except for the caste category. Most pregnant women were in the second trimester of pregnancy at enrolment. Maternal education, maternal age, stage of pregnancy, and pregnancy order were similar between the intervention and the control group. Household characteristics, wealth index, and poverty status were comparable in the intervention and control groups. The average wealth index score of participants from the intervention group (0.19, 95%CI 0.04–0.43) and the control group (0.18, 95%CI 0.07–0.45) was comparable (*p* = 0.965). At the endpoint, the mean wealth score was comparable to that at recruitment. Thus, the socio-economic status of the families was almost identical throughout the study period. Out of 656 newborns, 49.84% were boys, and 50.16% were girls.

**Table 1:** Baseline characteristics of study participants in the intervention and the control arm.

	Total (*n* = 656)	Intervention (*n* = 326)	Control (*n* = 330)	*p* value
*Maternal characteristics*				
*Age in years*; Mean (SD)	23.94 (3.61)	23.79 (3.57)	24.08 (3.64)	0.288
*Education*				
Illiterate	18 (2.74%)	11(3.37%)	7 (2.12%)	chi2 = 6.06 *p* = 0.195
Primary (1–5)	24 (3.66%)	12 (3.68%)	12 (3.64%)	
Secondary (6–10)	295 (44.97%)	156 (47.85%)	139 (42.12%)	
Higher Secondary	203 (30.95%)	100 (30.67%)	103 (31.21%)	
Graduate and more	116 (17.68 5)	47 (14.42%)	69 (20.91%)	
*Pregnancy duration*				
1st Trimester	132 (20.12%)	68 (20.86%)	64 (19.39%)	chi2 = 0.21 *p* = 0.64
2nd Trimester	524 (79.88%)	258 (79.14%)	266 (80.61%)	
*Gravida*				
First	182 (42.99%)	150 (46.01%)	132 (40%)	chi2 = 5.68 *p* = 0.224
Second	294 (44.82%)	145 (44.48%)	149 (45.15%)	
Third	64 (9.76%)	26 (7.98%)	38 (11.52%)	
Fourth	13 (1.98%)	4 (1.23%)	9 (2.73%)	
Fifth	3 (0.46%)	1 (0.31%)	2 (0.61%)	
*Total of live children*; Mean (SD)	0.59 (0.65)	0.61 (0.68)	0.58 (0.65)	*p* = 0.576
*Anaemia*				
No Anaemia	173 (30.40%)	82 (29.82%)	91 (30.95%)	chi2 = 2.43 *p* = 0.487
Mild Anaemia	203 (35.68%)	96 (34.91%)	107 (36.39%)	
Moderate Anaemia	191 (33.57%)	95 (34.55%)	96 (32.65%)	
Severe Anaemia	2 (0.35%)	2 (0.73%)	0	
*Father’s characteristics*				
*Age in years*; Mean (SD)	29.99 (4.13)	29.59 (3.63)	30.4 (4.54)	*p* = 0.012
*Education*				
Illiterate	21 (3.20%)	13 (3.99%)	8 (2.42%)	chi2 = 4.67 *p* = 0.322
Primary (1–5)	47 (7.16%)	23 (7.06%)	24 (7.27%)	
Secondary (6–10)	337 (51.37%)	175 (53.68%)	162 (49.09%)	
Higher Secondary	170 (25.91%)	82 (25.15%)	88 (26.67%)	
Graduate	81 (12.35%)	33 (10.12%)	48 (14.55%)	
*Household characteristics*				
*Caste category*				
Schedule Caste	52 (8.84%)	24 (8.11%)	28 (9.59%)	chi2 = 14.59 *p* = 0.002
Schedule Tribe	235 (39.97%)	141 (47.64%)	94 (32.19%)	
Backward classes	279 (47.45%)	123 (41.56%)	156 (53.42%)	
Open/General	22 (3.74%)	8 (2.70%)	14 (4.79%)	
*Wealth index*				
1st Quintile	110 (16.77%)	49 (15.03%)	61 (18.48%)	chi2 = 7.809 *p* = 0.099
2nd Quintile	122 (18.60%)	58 (17.79%)	64 (19.39%)	
3rd Quintile	143 (21.80%)	83 (25.46%)	60 (18.18%)	
4th Quintile	142 (21.65%)	75 (23.01%)	67 (20.30%)	
5th Quintile	139 (21.19%)	61 (18.71%)	78 (23.64%)	
*Average family size*; mean (SD)	4.66 (1.84)	4.86 (1.91)	4.47 (1.76)	*p* = 0.006
*Below poverty line*	286 (43.66%)	145 (44.62%)	141 (42.73%)	*p* = 0.626

Data are No (%); unless stated otherwise.

Table 2 compares the baseline characteristics of study participants lost to follow-up and those who completed the intervention. Sociodemographic characteristics, other than maternal education, were comparable between those who lost to follow-up and those who completed the intervention.

**Table 2:** Characteristics of study participants who lost to follow-up during the intervention at 24 months.

	Total (*N* = 656)	Retained (*n* = 588)	Loss to follow-up (*n* = 68)	*p* value
*Maternal characteristics*				
*Age in years*; Mean (SD)	23.94 (3.61)	24.02 (3.64%)	23.20 (3.23%)	0.076
*Education*				
Illiterate	18 (2.74%)	13 (2.21%)	5 (7.35%)	chi2 = 9.694 *p* = 0.046
Primary (1–5)	24 (3.66%)	20 (3.40%)	4 (5.88%)	
Secondary (6–10)	295 (44.97%)	261 (44.39%)	34 (50.00%)	
Higher Secondary	203 (30.95%)	186 (31.63%)	17 (25.00%)	
Graduate and more	116 (17.68%)	108 (18.37%)	8 (11.76%)	
*Pregnancy duration*				
1st Trimester	132 (20.12%)	111 (18.88%)	21 (30.88%)	chi2 = 5.465 *p* = 0.019
2nd Trimester	524 (79.88%)	477 (81.12%)	47 (69.12%)	
*Gravida*				
First	132 (40%)	112 (38.36%)	20 (52.63%)	chi2 = 6.937 *p* = 0.139
Second	149 (45.15%)	135 (46.23%)	14 (36.84%)	
Third	38 (11.52%)	35 (11.99%)	3 (7.89%)	
Fourth	9 (2.73%)	9 (3.08%)	0 (0.00%)	
Fifth	2 (0.61%)	1 (0.34%)	1(2.63%)	
*Anaemia*				
No Anaemia	173 (30.40%)	159 (30.93%)	14 (25.45%)	chi2 = 6.858 *p* = 0.077
Mild Anaemia	203 (35.68%)	189 (36.77%)	14 (25.45%)	
Moderate Anaemia	191 (33.57%)	164 (31.91%)	27 (49.09%)	
Severe Anaemia	2 (0.35%)	2 (0.39%)	0 (0%)	
*Father’s characteristics*				
*Age in years*; Mean (SD)	29.99 (4.13)	30.06 (3.96)	29.44 (5.36)	*p* = 0.241
*Education*				
Illiterate	21 (3.20%)	17 (2.89%)	4 (5.88%)	chi2 = 2.382 *p* = 0.666
Primary (1–5)	47 (7.16%)	41 (6.97%)	6 (8.82%)	
Secondary (6–10)	337 (51.37%)	304 (51.70%)	33 (48.53%)	
Higher secondary	170 (25.91%)	152 (25.85%)	18 (26.47%)	
Graduate	81 (12.35%)	74 (12.59%)	7 (10.29%)	
*Household characteristics*				
*Caste category*				
Schedule caste	61 (9.30%)	52 (8.84%)	9 (13.24%)	chi2 = 4.806 *p* = 0.187
Schedule Tribe	264 (40.24%)	235 (39.97%)	29 (42.65%)	
Backward classes	304 (46.34%)	279 (47.45%)	25 (36.76%)	
Open/General	27 (4.12%)	22 (3.74%)	5 (7.35%)	
*Wealth index*				
1st Quintile	110 (16.77%)	94 (15.99%)	16 (23.53%)	chi2 = 6.257 *p* = 0.181
2nd Quintile	122 (18.60%)	113 (19.22%)	9 (13.24%)	
3rd Quintile	143 (21.80%)	126 (21.43%)	17 (25%)	
4th Quintile	142 (21.65%)	125 (21.26%)	17 (25%)	
5th Quintile	139 (21.19%)	130 (22.11%)	9 (13.24%)	
*Average family size*; mean (SD)	4.66 (1.84)	4.47 (1.87)	4.68 (1.84)	*p* = 0.360
*Below poverty line*	286 (43.66%)	255 (43.44%)	31 (45.59%)	chi2 = 0.114 *p* = 0.735

Data are No (%); unless stated otherwise.

### Implementation milestones and quality

#### Home visits

We conducted 6,665 home visits throughout the intervention period. The preliminary review of first-quarter intervention data revealed that home visits were directive, 139 (23%) were family-centered. In 360 (69.62%) home visits, the average duration was less than expected, and 68 (11%) took more than 60 min. Despite rigorous training and certification, monitoring data and interactions in monthly meetings with the Balsakhi revealed low motivation and confidence to deliver interventions. Based on the findings and feedback, we re-designed the intervention delivery approach and introduced the community supervisors to retrain, handhold, and mentor the Balsakhi. We randomly supervised 1,670 (25%) home visits to ensure quality.

In the subsequent quarter, the indicator improved. Out of the total of 1,670 home visits supervised, 1,169 (70%) had interactive discussions, 1,458 (87.30%) used tools and other materials effectively during the session delivery, and in 295 (17.66%) home visits, male members from the household participated in the discussion and participation of male members increased from 4.3 to 32.65%. The coverage of the home visits also increased from 31.93% in the first quarter of intervention to 98.6% in the 6th quarter. Out of 294 households from the intervention group, 190 (64.63%) families received more than 75% of home visits, and 104 (35.37%) households received less than 75% of planned home visits (17). Data errors reduced from 270 to 140 per month on average.

#### Community workshops

In the intervention group, we conducted 25 toy workshops at the village level to support and train caregivers to prepare low-cost ECD play material from household items. The 295 participants trained to prepare and use a low-cost play material. Four demonstration centers were set up in each PHC area to demonstrate pregnant women and caregivers to prepare locally/socio-culturally acceptable recipes that meet mothers’ and child’s nutrition needs. A recipe book was prepared for complementary feeding and shared with the caregivers. 291 women/caregivers attended sessions in the demonstration center at least once.

#### Community meetings

We conducted 450 community meetings in the intervention villages over 24 months. Balsakhi (service provider) and outreach staff coordinated these meetings. Anganwadi workers, community members, mothers, and other care providers attended these meetings. These meetings allowed participants to reflect on their learnings, gaps, or challenges in service delivery and potential solutions.

#### Project staff monthly meetings

We held monthly meetings of Balsakhi (service providers) at each PHC. We conducted 65 Balsakhi meetings to share experiences and reflect on their learnings and challenges. The field supervisors presented the monthly coverage data to the stakeholders and discussed coverage gaps and opportunities to reach out to those who missed out in the previous months. The refresher training sessions were conducted during these meetings, whenever needed, to enhance the skills and competencies of the Balsakhi for conducting home visits. During these meetings, the collaborative re-design process associated with improved delivery indicators was agreed upon. The community and Project staff meetings helped improve the quality and coverage of the intervention. Another paper presents the data on improving the service delivery indicators (16).

#### Child and mother outcomes

The average weight gain for women from the intervention group was 9.01 (SD 3.74) kilogram weight, which was significantly more than the weight gain in women from the control group (7.67, SD 3.43) during pregnancy (*p* < 0.001). The intervention arm had a lower proportion of low birth weight newborns 68 (20.86%) than the control arm 88 (26.67%), but this difference was not statistically significant. However, the birth weight was significantly higher in the intervention arm 2.71 (SD 0.44) than in the control arm 2.61 (SD 0.45). Ninety-six percent of women started breastfeeding within 1 h of delivery and the exclusive breastfeeding rate was 89.80%.

Table 3 shows the statistically significant effect of the intervention on weight for height (WHZ) in children at 24 months. The effect was comparable for all other anthropometric indicators between the intervention and the control arm.

**Table 3:** Comparison of anthropometric indicators in the intervention and the control group.

Developmental domain	Intervention arm	Control arm	*p* value
	*n* = 303 at 12 months *n* = 296 at 24 months	*n* = 293 at 12 months *n* = 292 at 24 months	
*WAZ*			
12 months	−1.51 (1.12; −1.63. −1.38)	−1.57 (1.03; −1.69 −1.45)	0.493
24 months	−1.73 (1.01; −1.85 −1.62)	−1.78 (0.92; −1.89 −1.67)	0.515
*HAZ*			
12 months	−0.94 (1.28; −1.08. −0.79)	−0.97 (1.33; −1.12 −0.82)	0.778
24 months	−1.37 (1.09; −1.49 −1.24)	−1.21 (1.07; −1.34 −1.09)	0.085
*WHZ*			
12 months	−1.36 (1.28; −1.51. −1.21)	−1.47 (1.2–1.61 −1.33)	0.286
24 months	−1.47 (1.13; −1.60 −1.34)	−1.66 (1.07; −1.78 −1.54)	0.033

Data are mean (SD; 95%CI). The analysis is adjusted for the cluster & assessors and controlled for covariates (maternal age, maternal education, wealth index, poverty status, caste category, gravida and maternal anaemia) by a mixed-effect model.

We observed a small effect on cognitive, language, motor, and socio-emotional development at 12 months. A difference in the mean child development outcome scores at 12 months between the intervention and the control group was not statistically significant (*p* > 0.05). The effect sizes increased for cognitive (Cohens *d* = 0.31; 95% CI: 0.13–0.48), language (Cohens *d* = 0.2; 95% CI: 0.01 0.39), motor (Cohens *d* = 0.27; 95% CI: 0.11–0.43); socioemotional development (Cohens *d* = 0.19; 95% CI: 0.01 0.37) at 24 months compared to 12 months of intervention. The intervention had a statistically significant effect on child development outcomes between the intervention and the control arm at 24 months (*p* < 0.05; Table 4).

**Table 4:** Comparison of child development outcome, mother–child interaction and home environment scores in the intervention and the control group.

Developmental domain	Intervention arm	Control arm	Value of *p*
	*n* = 303 at 12 months *n* = 296 at 24 months	*n* = 293 at 12 months *n* = 292 at 24 months	
*Cognitive*			
12 months	56.91 (8.84; 55.91–57.91)	56.29 (10.1; 55.13–57.44)	0.239
24 months	70.18 (8.71; 69.18–71.18)	67.65 (9.70; 66.53–68.77)	0.001
*Language*			
12 months	9.03 (3.10; 8.68–9.38)	8.84 (3.38; 8.45–9.23)	0.117
24 months	18.25 (4.86; 17.70–18.81)	17.41 (5.35; 16.79–18.03)	0.002
*Motor*			
12 months	38.36 (6.63; 37.61–39.11)	38.34 (7.32;37.49–39.18)	0.042
24 months	53.36 (5.21; 52.76–53.96)	51.97 (6.01; 51.28–52.67)	0.046
*Socioemotional*			
12 months	20.29 (6.54; 19.55–21.03)	20.37 (6.98; 19.56–21.17)	0.443
24 months	21.47 (4.86; 20.88–22.07)	20.51 (5.62; 19.86–21.16)	0.031
*Home inventory*			
12 months	37.06 (4.61; 36.54–37.58)	35.65 (5.05; 35.06–36.23)	0.035
24 months	36.58 (5.21; 36.08–37.06)	35.61 (4.65; 35.07–36.14)	0.008
*Mother–child interaction*			
12 months	36.01 (7.34; 35.17–36.83)	34.54 (7.63; 33.66–35.42)	0.014
24 months	40.37 (5.42; 39.75–40.99)	38.2 (6.01; 37.50–38.89)	<0.001

Data are mean (SD; 95%CI). The analysis is adjusted for the cluster & accessors and controlled for covariates (maternal age, maternal education, wealth index, poverty status, caste category, gravida and maternal anaemia) by a mixed effect model.

**Figure 2:**
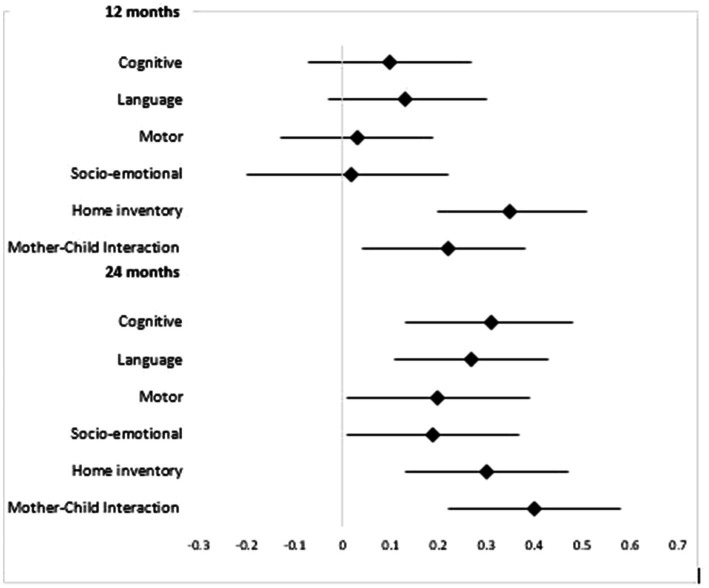
Effect size (Cohens *d*) with 95%CI of intervention on child development domains at 12 and 24 months.

The intervention statistically impacted the home environment and mother–child interaction at 12 months (Figure 2). We observed moderate to mild but statistically significant effects on the home environment at 24 months (0.3, 95% CI: 0.05–0.43). The effect of the intervention was maximum for mother–child interaction at 24 months (0.4, 95% CI, 0.22–0.58; Figure 2).

The number of children with improved scores in the intervention group at 24 months was more compared to 12 months for the cognitive domain (181, 61.15% versus 157, 51.82%, *p* = 0.021) and motor domain (224, 75.68% versus 139, 45.87%, *p* < 0.001). However, the difference was not statistically significant for language and socioemotional domains.

The lowest wealth quantile shows the maximum and statistically significant impact of the intervention on cognitive development (Cohens *d* = 0.92; 95% CI = 0.53–1.30), motor development (Cohens *d* = 0.72; 95% CI = 0.29–1.14); language development (Cohens *d* = 0.79; 95% CI = 0.43–1.16). The intervention had a lower effect on other wealth quantiles. Study participants below the poverty line as per the Indian Government’s categorization had a statistically significant minimal to moderate effect size on cognitive (Cohens *d* = 0.43; 95%CI = 0.19–0.67), motor (Cohens *d* = 0.19; 95% CI = 0.03–0.42), language (Cohens *d* = 0.25; 95% CI = 0.02–0.49) and socio-emotional development (Cohens *d* = 0.36; 95% CI = 0.10–0.62) (Supplementary Table S1).

## Discussion

Our study also demonstrated that the effectiveness of a responsive parenting program integrated with nutrition intervention significantly affected the development of children under 2 years of age and promoted a conducive home environment and mother-to-child interactions. The optimum growth and development of children under three may break the cycle of inequality and vulnerability and lay the foundation for achieving sustainable development goals (22, 31).

We co-created and implemented a responsive parenting and nutrition program delivered through community networks that included Government Anganwadi Centers for 24 months. The findings emphasized that community engagement, a theory-driven conceptual framework, and formative research are needed to design and implement complex interventions effectively. A paper by Bentley and colleagues also stressed a need to contextualize a program through an inclusive process and sustained stakeholder engagement to improve the quality of delivery (32).

Our program’s key highlight was sustained community engagement. A ‘Balsakhi,’ meaning the friend of a child in the local language, delivered the entire intervention. Despite the well-conceptualized intervention, which draws upon the community’s strengths and is contextually appropriate, we faced challenges in intervention delivery by the community volunteers in the initial stages. Quality of service delivery was a primary concern, and data revealed coverage gaps, including community volunteers’ low motivation and engagement. Thus, we redesigned the implementation approach to motivate and engage service providers to deliver the intervention with fidelity. We appointed a community supervisor for handholding and mentoring community volunteers. Supervisors accompanied the community volunteers in at least 25% of the home visits. The implementation data revealed that the coverage improved over time and significantly improved the quality of intervention delivery. We presented the data in a separate paper, (16). We adopted a data-driven approach to improve coverage and quality of intervention delivery, and changes in practice developed out of the evidence shared across the network of stakeholders. Thus, our study emphasized that engaging the remotest field staff, community members, and other stakeholders in the data review and decision-making process motivates and improves their engagement, creates an ecosystem that improves accountability and efficiency, and empowers everyone involved. If a community volunteer is mentored, supported, and monitored, they can deliver the complex integrated intervention in early childhood with the desired fidelity.

Our program included fortnightly home visits along with monthly group sessions. Our trial’s frequency of contact with caregivers was similar to earlier studies from India (33) and Bangladesh (34, 35). However, it was less than earlier Jamaican trials, which reported weekly play sessions through home visits (36–39). Even though the group-based parenting education programs are practical and potentially cost-effective options (40), we decided to adopt a combination of methods, both the home visits and group sessions, based on the local context and caregivers’ needs. Our approach of home visits to tailor the intervention to caregivers’ specific needs, while group sessions facilitated peer learning through experience sharing, is supported by the evidence. Group sessions enable ECD culture across the community, and the home visit strengthens family processes (40, 41).

Our data showed a positive and statistically significant impact on the home environment and mother–child interaction. Mothers from the intervention group showed improved knowledge and skills for responsive parenting. Our study substantiates the findings of a systematic review that parenting interventions improve parenting knowledge, skills, parent–child interactions, and home environment are the critical pathways to bringing positive change in child development (42).

The intervention had a maximum effect on Cognitive development, followed by language, motor, and socio-emotional development. Our results were comparable to the systematic review by Jeong and colleagues, which included 102 studies from 33 countries, concluding that parenting interventions in the first 3 years of life improve a child’s cognitive, language, motor, and socioemotional development and reduce behavioral problems (42).

We observed that these effects on child developmental outcomes increased over time. Assessing the impact of the duration of the intervention on child development outcomes was not the primary objective of this trial; however, our data highlighted that the intervention given for a longer duration, that is 24 months, shows more benefits than an intervention delivered over 12 months. A systematic review published in 2021 reported a lack of evidence on the effect of variable program duration on child development outcomes (38). Further study is needed to separate the influence of age at assessment from that of the duration of the intervention. It would be pertinent to enroll families for variable periods to explore the benefits over time and follow up a long-term cohort to understand how intervention benefits can be sustained beyond 24 months, even up until adolescence.

One of the limitations of our study is that we should have included nutrition supplementation. However, to avoid duplication of services offered at the Anganwadi Centers under the current ICDS program of India, we linked the beneficiaries to the Anganwadi Centers for nutritional supplementation. In previous studies, direct nutrition supplementation within a parenting intervention has shown significant benefits for 24 months of child age (34, 43). The integrated nutrition components in our study focused on information and practical ideas. The effective delivery of nutrition messages is essential, but more is needed. Nutrition-specific and sensitive interventions are needed, which include food security issues to improve feeding behaviors, the sufficiency and quality of complementary foods, maternal nutrition (preconception and during pregnancy), and birth spacing (45, 46). These issues were beyond the scope of this study. Another major challenge was that the families found difficulty maintaining vegetable gardens in April and May due to intense heat in the region and water scarcity. Our study suggests these actionable components require further contextualization to embed more firmly into local practices for sustainability and scalability.

Our program has several strengths. Despite the strong evidence from neurosciences and economics regarding the benefit of the intervention in the early years, most parenting interventions for cognitive and behavioral development are targeted at older children, at preschoolers (42–46). Our study recruited pregnant women, and the intervention continued till 24 months of the child’s age, providing evidence of impact and engagement at the foundational stages of growth. Another strength of the study is an integrated intervention delivery through community-led channels. In resource-constrained settings, such an approach may be cost-effective. In addition to program-level advantage, available evidence suggests no significant loss in effect size when intervention is delivered in community settings through community volunteers (12).

In addition to the evidence of the combined effect of nutrition and responsive parenting programs on child development, our study has provided program and delivery level guidance to positively influence interventions’ quality, engagement, and sustainability. The needs-based approach we employed guides the rapid re-design of delivery mechanisms, which was associated with acceptance by the community and led to a shift in responsibility and accountability at a local level (22). To further understand effective delivery mechanisms, rather than purely focusing on assessing responsive parenting in the mother, we recommend future studies that consider others in the support network for ECD (47), such as older siblings, grandparents, and other relatives, who can play a more prominent role on ECD in extended family or joint families structures.

The strength of our innovation is that it aligns with the recommendation of 2013 National Early Childhood Education and Care Policy of the Government of India, enhancing the potential for sustainable scaling. Our innovation was designed to address scalability and replicability, to establish self-sustaining village-level units that serve as models for neighboring communities, and to foster expansion. To ensure the sustainability and expansion of our program, we prioritized community engagement and ownership, engaging local stakeholders, parents, and community leaders in the design and implementation of our intervention. We optimized resource utilization by leveraging locally local assets and investing in capacity building, thereby reducing reliance on external funding. A robust monitoring and evaluation system built around shared accountability, ensures continuous assessment. Regular review of effectiveness and impact of the program enables data-driven adjustments to meet evolving needs. This comprehensive approach ensures our program’s lasting impact and continued expansion of the innovation, benefiting a wider population of children, their families and communities.

To conclude, our study emphasized the importance of developing a conceptual framework integrating a theoretical model with formative research for designing and redesigning a complex intervention. The study provides an evidence-based, responsive curriculum, with implementation strategies grounded in social learning theory that enhance caregivers’ knowledge and skills for promoting early child development. Due to the pragmatic nature of the study, our intervention also has the potential to integrate within the existing Integrated Child Development Program in India.

## Data availability statement

The original contributions presented in the study are included in the article/Supplementary material, further inquiries can be directed to the corresponding author.

## Ethics statement

The studies involving humans were approved by Institutional Ethics Committee of Datta Meghe Institute of Medical Sciences (Deemed to be University) approved the trial vide letter with Ref no: DMIMS (DU)/IEC/2017-18/6306 dated 27.03.2017. The studies were conducted in accordance with the local legislation and institutional requirements. The participants provided their written informed consent to participate in this study.

## Author contributions

ZQS and AG conceptualized the study and led the design as the primary author, analyzed data, and led the write-up. ST developed the data collection materials with inputs from ZQS, AG, and PH. PH and MNK oversaw the study, data analysis, and interpretation, and drafted the manuscript. PK and MP trained and supervised the data collection team. ST and AG oversaw the quality assurance. MP administrated the project work. SG, PK, PH, DS, and SC assisted with the write-up and participated in the study design, data analysis, and interpretation. All authors contributed to the article and approved the submitted version. All authors critically reviewed drafts of the manuscript.
