# Supplementary material for: Design-redesign, implementation, and evaluation of effectiveness of maternal nutrition and responsive parenting program on child development at 2 years of age from rural India: a cluster RCT
Source: Front Public Health. 2023 Nov 14;11:1165728. doi: 10.3389/fpubh.2023.1165728 (PMC10682778; doi:10.3389/fpubh.2023.1165728)
Supplement: Supplementary file 1 [file Table_1.DOCX]

**Supplementary File**

1. **Quality Indicators:**

- **Process Indicators**
- % of visits as per the scheduled time
- % of visits with duration of visit 1) < 15 min 2) 15 to 30 min 3) >30 min
- Average duration of visits
- % of visits where parents asked any questions
- % of visits done by Balsakhi alone
- % of visits of Balsakhi accompanied by the supervisor
- % of households received complete intervention segregated for male & female
- % of vulnerable families/ Household received the complete intervention
- No. of services delivered to ANC and to PNC
- **Outcome Indicators**
- Average weight gain during pregnancy
- % of women started breastfeeding within 1 hr for caesarean and half hour for normal delivery
- % of women delivered a baby having birth weight <2500gm and >=2500gm
- **Coverage**
  - Actual Visits done*100/No. of planned visits segregated Village wise, ANC/PNC, Kitchen Garden & SHG
  - Coverage of FRO visits and supervisory visits in a week
  - Line list of beneficiaries missed out from the previous week covered in the current week
  - Line list of beneficiaries who missed out during the week
  - Complete and Timely reporting of data

1. **Monitoring, Evaluation, and Learnings (MEL)**

We adopted the principles of monitoring, evaluation, and learning to guide the overall project implementation to ensure the coverage of the program and fidelity of data to make the real-time decision and evidence adaptation of the program to the felt need. We integrated our MEL system with the Theory of Change (TOC) model. Our MEL system is based on five aspirations. The five aspirations describe additional, interconnected, concepts that together suggest that practitioners and researchers within ECD use data, decision making, monitoring, evaluation, and learning in a way that strives to be: Inclusive, Dynamic, Informative, Interactive, and people-centered. We designed the “ANC/PNC Visit Diary” for recording the delivered intervention at home visits and Balsakhi diaries for detailing the narrative feedback of the beneficiary family and relevant comments on the home environment. Also, each visit report of Balsakhi/FRO was to be signed by the person/woman attending the home visit session. This helped to ensure the reach and delivery of intervention with the required fidelity and quality. Our system encompasses the indicators mentioned above.

1. **Intervention Management System**

We developed a good system for intervention delivery and data management including for Balsakhi to keep the records in hard copies. The intervention management system of ongoing data collection and almost real-time analysis will help us to take timely and evidence-based decisions to enhance the quality and coverage of the intervention. A similar system we developed for the other components of the intervention as well. The daily data collected by peer mentor/Balsakhi will enter weekly into a tablet. We use ODK collect (open source) to generate electronic forms. Data is pushed to the server on the same day, the next day it is extracted and analysed for the predefined key process indicators. We use STATA for quality checking and the quality of data is discussed in the weekly and monthly review meetings. The whole process is continuously ongoing.


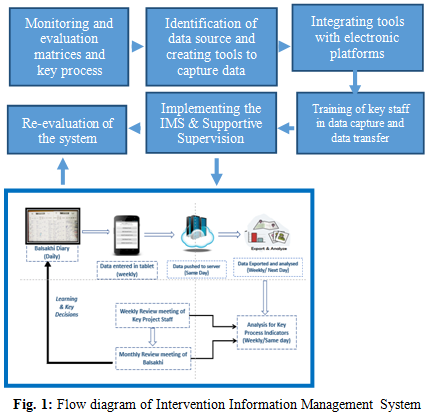


A calendar of home visits is prepared for each pregnant woman as per the date of LMP and EDD. After delivery, the EDD in the calendar is replaced by the date of delivery, thus the calendar is updated as per the date of delivery. Thus, each mother-child dyad is expected to receive a maximum of 39 home visits if the pregnancy was registered at the end of the first trimester (3 months of gestation). These individualized visit calendars are provided to the corresponding Balsakhis and FRO with a list of pregnant women in their service area. The home visits include well-defined activities as per the stage of pregnancy and child age.

1. **Intervention Description:**

The intervention is being delivered through the following component:

1. Fortnightly home visits for targeted intervention
2. Caregivers Support Group Meetings/sessions
3. Community Meetings & sensitization programs at village levels
4. Low-cost toy/play material making workshops
5. Nutrition demonstration Center
6. Nutrition Garden where feasible

**Fortnightly home visits:** The parenting intervention was designed to provide a variety of play and communication activities using everyday household items or homemade toys to help caregivers to stimulate children’s cognitive, language, motor, socio-emotional skills, and physical development.

**Caregivers Support Group Meetings/sessions:** Balsakhi and MTS with support from AWW jointly organized caregivers support group meeting at respective AWC. The purpose of this meeting was to provide an opportunity for participants to share what they have learned from the home visit sessions, reflect on important gaps and challenges.

**Community Meetings & sensitization programs:** The purpose of the sensitization program was to promote community engagement and create a conducive atmosphere for holistic childhood development, the first 1000 days of a child’s life.

**Low-cost toy/play material-making workshops:** The purpose of toy making workshop was to support and train caregivers to prepare the low-cost ECD play material from whatever is available in the household. The workshops also help parents to understand how to use the toys/play material, what development domain it addresses, and what to observe while using the toys/material.

**Nutrition demonstration Center:** The main goal was to ensure the availability and sustainability of locally/socio-culturally acceptable nutrition for pregnant mothers and children in every household. It includes setting up some demonstration units and educational for nutritional training programs in the community.

**Nutrition Garden:** To provide nutritional supplements to pregnant and lactating mothers and children. Nutrition gardens in households, were feasible at village level. We have provided seeds/saplings and training to potential households, whereas the household has to provide adequate space, and other resources like water, etc. The Stepping Stones project promoted environment-friendly low-cost organic food growing techniques and discourages the use of chemical-based fertilizers.
