# Supplementary material for: Design-redesign, implementation, and evaluation of effectiveness of maternal nutrition and responsive parenting program on child development at 2 years of age from rural India: a cluster RCT
Source: Front Public Health. 2023 Nov 14;11:1165728. doi: 10.3389/fpubh.2023.1165728 (PMC10682778; doi:10.3389/fpubh.2023.1165728)
Supplement: Supplementary file 2 [file Table_2.docx]

**Supplementary File**

**Table S1: Effect of intervention in various population sub group at 24 months of age**

|  | **Cognitive** | | **Motor** | | **Lang** | | **PSED** | |
| --- | --- | --- | --- | --- | --- | --- | --- | --- |
|  | **effect size (95% CI)** | **p value** | **effect size (95% CI)** | **p value** | **effect size (95% CI)** | **p value** | **effect size (95% CI)** | **p value** |
| **Wealth Quintiles** |  |  |  |  |  |  |  |  |
| **Lowest** | 0.92  (0.53 1.30) | 0.000 | 0.72  (0.29 1.14) | 0.001 | 0.79 (0.43 1.16) | 0.000 | 0.18 (-0.28 0.64) | 0.442 |
| **Lower-Middle** | 0.22  (-0.14 0.60) | 0.239 | 0.13 (-0.27 0.52) | 0.527 | 0.12 (-0.23 0.47) | 0.491 | 0.28 (-0.08 0.64) | 0.134 |
| **Middle** | 0.31  (-0.01 0.63) | 0.058 | 0.22 (-0.12 0.55) | 0.209 | 0.18 (-0.17 0.54) | 0.317 | 0.36 (0.03 0.69) | 0.029 |
| **Higher-Middle** | - 0.08  (-0.44 0.27) | 0.648 | 0.16 (-0.28 0.60) | 0.479 | -0.03(-0.4 0.33) | 0.859 | minus 0.01 (-0.38 0.34) | 0.928 |
| **Highest** | 0.29  (-0.10 0.70) | 0.145 | 0.14 (-0.15 0.43) | 0.344 | 0.07 (-0.30 0.46) | 0.692 | 0.21 (-0.15 0.56) | 0.257 |
|  |  |  |  |  |  |  |  |  |
| **Sex** |  |  |  |  |  |  |  |  |
| **Male** | 0.32 (0.11 0.54) | 0.003 | 0.26 (0.01 0.51) | 0.039 | 0.22 (0.01 0.44) | 0.044 | 0.22 (-0.02 0.46) | 0.074 |
| **Female** | 0.35 (0.08 0.62) | 0.010 | 0.30 (0.07 0.53) | 0.01 | 0.21 (-0.08 0.51) | 0.158 | 0.27 (0.03 0.52) | 0.024 |
|  |  |  |  |  |  |  |  |  |
| **Below poverty line (BPL)** |  |  |  |  |  |  |  |  |
| **yes** | 0.43 (0.19 0.67) | 0.000 | 0.19 (-0.03 0.42) | 0.090 | 0.25 (0.02 0.49) | 0.032 | 0.36 (0.10 0.62) | 0.006 |
| **no** | 0.21 (-0.03 0.46) | 0.096 | 0.33 (0.10 0.56) | 0.004 | 0.17 (-0.08 0.43) | 0.181 | 0.06 (-0.19 0.32) | 0.625 |
